# Noninvasive Assessment of Arterial Compliance and Lumen Pressure in Human Carotid Arteries Using Physics-Informed Neural Networks and Ultrasound Imaging: A Clinical Feasibility Study

**DOI:** 10.21203/rs.3.rs-10280788/v1

**Published:** 2026-07-16

**Authors:** Tuhin Roy, Parth Gami, Elisa E. Konofagou

**Affiliations:** 1 Dept. of Biomedical Engineering, Columbia University, New York City, USA;; 2 Dept. of Radiology (Physics), Columbia University, New York City, USA;; 3 Dept. of Neurosurgery, Columbia University, New York City, USA;

**Keywords:** Arterial compliance, Pulse wave imaging, Vector flow imaging, Inverse problem

## Abstract

Arterial stiffness is an independent predictor of cardiovascular mortality, with localized compliance variations providing key insights for vascular disease assessment. In our previous studies, we implemented a PINN-based inverse approach integrating Pulse Wave Imaging (PWI) and Vector Flow Imaging (VFI) data with 1D PDEs of pulse wave propagation and was trained to minimize mismatch between measured data and physics constraints for *in silico* and phantom-mimicking stenotic vessels. This study evaluates the performance of the previously-reported PINN framework for noninvasive, spatially resolved estimation of arterial compliance and lumen pressure from the high-frame-rate ultrasound (PWI and VFI) data in a clinical setting. The framework was applied on common carotid artery high-frequency ultrasound frames (3 kHz frame rate) in five subjects: one healthy and four with carotid stenosis (low to medium occlusion). The proposed model estimated spatially varying compliance in all subjects: healthy subjects showed uniform compliance, while stenotic subjects exhibited focal compliance variation correlating with plaques/occlusion on ultrasound B-mode images. The model reconstructed wall motion (% difference <0.2% in healthy, <0.48% in carotid stenosis patients) and flow velocity (% difference <0.2% in healthy). In stenotic cases, the model adapted to unreliable flow data by using the wall motion data only. The computation time ranged from 20 minutes to 1.5 hour depending on the spatial lateral resolution; lateral resolution of 16-elements data (instead of full 128-elements data of L7–4 transducer) considered for healthy cases required 20 minutes runtime, whereas, for the stenotic cases, 1.5 hours was needed due to the required full resolution (all 128-elements data). In conclusion, this study demonstrates the effectiveness of the PINN-based framework for non-invasive, patient-specific mapping of localized arterial compliance variation as well as luminal pressure. These findings presented in this feasibility study support clinical translation for non-invasive atherosclerotic plaque risk evaluation and personalized cardiovascular assessment.

## Introduction

Cardiovascular disease (CVD) remains the leading cause of global mortality. Among the risk factors for CVD, arterial stiffness has emerged as a powerful and independent predictor of cardiovascular morbidity and mortality [1,2]. Increased arterial stiffness elevates systolic blood pressure, imposes excessive left-ventricular afterload, and compromises diastolic coronary perfusion, thereby creating a mechanistic link between vascular aging and adverse cardiac outcomes. Epidemiological studies have consistently demonstrated that arterial stiffness, as quantified by Pulse Wave Velocity (PWV) [3], is associated with an increased risk of myocardial infarction, stroke, heart failure, and all-cause death, independent of traditional cardiovascular risk factors. This predictive capability extends not only to high-risk populations but also to asymptomatic individuals without overt cardiovascular disease, suggesting that arterial stiffness provides early insights into vascular health and offers opportunities for timely intervention.

While global measures of arterial stiffness, such as carotid-femoral PWV obtained via applanation tonometry, have proven valuable in risk stratification, they inherently provide only spatially averaged estimates of vascular properties over the entire circulation [4]. However, many common vascular pathologies including atherosclerotic plaques, carotid stenosis, and abdominal aortic aneurysms induce localized, heterogeneous changes in arterial mechanical properties [5,6]. Atherosclerotic plaques in the carotid artery, for instance, are a major source of ischemic stroke, with approximately one-fifth of strokes originating from carotid plaque rupture and subsequent distal embolization [7]. Importantly, the vulnerability of a plaque to rupture is determined not solely by the degree of luminal stenosis, but critically by its composition and mechanical properties [5,6]. Plaques with large lipid-rich necrotic cores, thin fibrous caps, and intraplaque hemorrhage - collectively termed “vulnerable plaques” - are prone to rupture even in the absence of severe stenosis. Therefore, localized, spatially resolved assessment of arterial compliance and stiffness is essential for accurate characterization of vascular disease and for predicting rupture risk in atherosclerotic lesions and aneurysms.

Traditional imaging modalities for assessing carotid plaque vulnerability include magnetic resonance imaging (MRI) and ultrasound. MRI is considered the gold standard for detecting features associated with plaque instability, such as intraplaque hemorrhage and lipid content [8–10]. However, MRI is limited by time constraints, contraindications in certain patient populations, cost [11], and a maximum *in vivo* spatial resolution of approximately 0.7 mm even with dedicated coils [12]. In contrast, for carotid plaques applications, ultrasound offers at least twice the spatial resolution of MRI, is widely available in clinical settings, is noninvasive, and is well-suited for real-time bedside assessment [11]. Recent advances in ultrasound-based elastography techniques, particularly shear wave elastography, have enabled direct mechanical characterization of arterial walls and plaques by inducing shear waves via acoustic radiation force or external vibration and tracking shear wave propagation to infer tissue stiffness [13,14]. While effective for straight arteries [15,16], shear wave elastography faces challenges in stenotic and highly calcified arteries, where reduced displacement amplitudes and complex wave interactions result in low signal-to-noise ratios and unreliable stiffness estimates [17–19].

An alternative ultrasound-based approach, Pulse Wave Imaging (PWI) [20–26], leverages the natural pulsatile motion of the arterial wall induced by the cardiac cycle to visualize pulse wave propagation and map regional pulse wave velocity along the vessel. PWI is noninvasive, requires no external excitation, and has been shown to differentiate plaque stiffness constituents in phantom experiments [27,28]. Complementary to PWI, Vector Flow Imaging (VFI) [29,30] provides two-dimensional blood flow velocity information by tracking blood scatterers using high-frame-rate speckle tracking and singular value decomposition filtering to separate wall motion from flow. While PWI and VFI provide rich spatiotemporal data on wall motion and blood flow, translating these measurements into quantitative estimates of arterial compliance; a fundamental biomechanical property intrinsically linked to wall stiffness and independent of boundary conditions remains a significant challenge. Analytical models, such as the Moens-Korteweg equation [31], relate PWV to wall stiffness under assumptions of homogeneity, straight infinite geometry which are violated in diseased arteries. Additionally, for the heterogeneous arteries, the estimation of localized variation of the arterial wall stiffness is required.

To address these limitations, researchers have formulated the estimation of arterial mechanical properties as an inverse problem, in which computational models of pulse wave propagation are iteratively adjusted to fit measured data, thereby recovering spatially varying compliance or stiffness distributions. Early inverse approaches, such as the Pulse Wave Inverse Problem (PWIP) framework [32], employed finite-difference methods to solve the governing partial differential equations (PDEs) describing one-dimensional pulse wave propagation, coupled with gradient-based optimization to minimize the discrepancy between model predictions and measured wall motion. While effective, these methods require precise knowledge of inlet and outlet boundary conditions - quantities that are difficult or impossible to measure noninvasively in clinical settings, and can be sensitive to measurement noise and model parameter initialization.

In recent years, physics-informed neural networks (PINNs) have emerged as a transformative paradigm for solving forward and inverse problems in science and engineering, including biomedical applications [33,34]. PINNs represent a hybrid approach that combines the expressive power of deep learning with the interpretability and physical consistency of governing equations. Specifically, PINNs parameterize the solution of PDEs using a forward-feed neural network and enforce adherence to the governing equations by incorporating the PDE residuals as a physics-based regularization term in the loss function. This formulation offers several key advantages over conventional numerical methods: (1) PINNs are mesh-free, eliminating the need for spatial discretization and simplifying the treatment of complex geometries; (2) PINNs can learn from sparse, noisy data by leveraging the physics constraints to interpolate and extrapolate beyond observed measurements; (3) PINNs are robust to unknown boundary conditions, as the physics residual is enforced throughout the entire spatiotemporal domain rather than relying on explicit boundary specifications; and (4) PINNs enable simultaneous state and parameter estimation, allowing for the direct recovery of unknown material properties (e.g., compliance) alongside the full spatiotemporal fields of pressure, flow velocity, and wall motion.

The application of PINNs to cardiovascular biomechanics is particularly promising. Recent studies have demonstrated the feasibility of using PINNs to predict arterial blood pressure from noninvasive 4D flow MRI data, to model cardiac electrophysiology and hemodynamics [35], and to characterize soft tissue mechanical properties in patient-specific geometries [36]. In the context of arterial stiffness estimation, our group previously developed a PINN-based inverse framework that integrates wall displacement and blood flow velocity data from PWI and VFI with linearized one-dimensional PDEs governing pulse wave propagation in heterogeneous vessels [37–39]. This framework was validated through *in silico* simulations mimicking homogeneous and stenotic arteries with plaques of varying stiffness, as well as through physical experiments using polyvinyl alcohol (PVA) phantoms with embedded plaque-like inclusions. The results demonstrated that the PINN model effectively captured localized variations in arterial compliance, remained robust under varying inlet and outlet boundary conditions, and achieved improved accuracy when both wall motion and flow velocity data were incorporated compared to wall-only methods.

While the results from *in silico* and phantom studies [39] were promising, the translation of the PINN-based inverse framework to *in vivo* human data represents a critical and necessary step toward clinical applicability. *In vivo* human arteries present unique challenges not encountered in controlled simulations or phantom experiments, including unknown and time-varying boundary conditions, natural cardiac waveforms with inter-subject variability, patient-specific anatomical heterogeneity, the presence of surrounding soft tissue that may induce acoustic aberrations, and complex flow patterns in stenotic regions where the assumptions of one-dimensional flow may be violated.

Thus, the objective of this study is to assess the clinical feasibility of the PINN-based inverse framework for estimating spatially varying arterial wall compliance and luminal pressure in human common carotid arteries using high-frame-rate ultrasound data. Specifically, we applied the previously validated PINN model to PWI and VFI data acquired from a healthy adult volunteer and from four patients with varying degrees of carotid artery stenosis. We hypothesized that the PINN framework would successfully recover localized compliance variations corresponding to visible atherosclerotic plaques on B-mode imaging, and demonstrate robustness to missing or unreliable flow data in stenotic cases. By establishing the feasibility of the PINN approach in human subjects, this work lays the foundation for future clinical applications in risk stratification of atherosclerotic plaques, prediction of plaque rupture, and personalized assessment of vascular health.

In the following sections, we describe the study population, the ultrasound data acquisition protocol, the PWI and VFI processing pipelines, the PINN model architecture and training procedure, and the results obtained in healthy and stenotic subjects. We then discuss the results and clinical implications of our findings, the current limitations of the approach, and future directions for advancing this technology toward clinical use in the assessment of carotid artery disease and stroke risk.

## Methods

### Study Design and Participants

This clinical study was conducted under a protocol approved by the Institutional Review Board (IRB) of Columbia University (protocol number AAAR0022). All participants provided written informed consent prior to enrollment. The study cohort consisted of five subjects: one healthy subject without known cardiovascular disease and four patients with carotid artery stenosis (details are in Table 1).

### Ultrasound Data Acquisition

High-frame-rate ultrasound channel data were acquired using a Verasonics Vantage 256 research ultrasound system (Verasonics Inc., Kirkland, WA, USA) and a Philips L7–4 linear array transducer (Philips Healthcare, Andover, MA, USA). The transducer consisted of 128 elements operating at a center frequency of 5.2 MHz with a bandwidth of 4–7 MHz. This configuration provided adequate penetration depth and spatial resolution for visualizing the common carotid artery (CCA) and capturing wall motion and blood flow dynamics.

Participants were positioned supine with the neck extended and rotated slightly away from the side of interest to optimize acoustic access to the CCA. The transducer was placed longitudinally over the CCA. Ultrasound gel was applied to ensure adequate acoustic coupling. Plane wave imaging was employed with coherent compounding across three transmission angles (−2°, 0°, +2°) to enhance image quality while maintaining a high effective frame rate. The pulse repetition frequency (PRF) was set to 8333 Hz, yielding a compounded frame rate of approximately 3 kHz after angular compounding. Radiofrequency (RF) channel data were recorded continuously over multiple cardiac cycles (typically 2–3 cycles) to ensure adequate temporal sampling of pulse wave propagation and blood flow patterns.

### Pulse Wave Imaging (PWI)

The PWI pipeline was used to extract arterial wall motion from the beamformed RF data. Following data acquisition, delay-and-sum beamforming was applied to the channel data to reconstruct full RF frame sequences. This beamforming step was GPU-accelerated to handle the large data volumes generated by high-frame-rate imaging. The anterior and posterior walls of the CCA were manually segmented on B-mode images to define regions of interest for motion tracking.

To estimate wall displacement, a GPU-accelerated one-dimensional normalized cross-correlation (NCC) algorithm was applied to consecutive beamformed RF frames. This approach achieved subsample displacement accuracy by employing quadratic interpolation around the correlation peak. Axial wall velocities were computed by temporal differentiation of the displacement time series. To eliminate rigid body motion artifacts (e.g., from breathing or probe movement), the velocity of the posterior wall was subtracted from that of the anterior wall, yielding the relative wall distension velocity.

Pulse wave velocity (PWV) was estimated using a linear regression approach applied to the time-of-arrival of the peak wall acceleration (time-derivative of the wall velocity) at different spatial locations along the imaged arterial segment, as described in prior studies. This provided a localized estimate of arterial stiffness for comparison with the spatially resolved compliance maps generated by the PINN model.

### Vector Flow Imaging (VFI)

The VFI framework was employed to quantify two-dimensional blood flow velocity within the carotid lumen. Blood flow was estimated by estimating the motion of blood scatterers using 2D normalized cross-correlation (NCC) techniques applied to the beamformed RF data [30]. To isolate blood flow from wall motion, singular value decomposition (SVD) filtering was performed on the spatiotemporal RF data matrix. Specifically, the low-rank components of the SVD, corresponding to slow-moving wall tissue, were discarded, while the higher singular values, corresponding to faster-moving blood scatterers, were retained for flow estimation.

Following SVD filtering, a 2D NCC kernel was applied in a two-dimensional search space (axial and lateral directions) to estimate inter-frame displacements of the blood scatterers. The NCC kernel had an underlying size of 1.4784 mm with an axial window overlap to ensure dense spatial coverage [30]. Inter-frame displacements were then normalized by the frame interval to obtain axial and lateral flow velocity components. For the purposes of the one-dimensional PINN model described below, only the lateral (along-vessel) flow velocity component was retained, and specifically, the peak systolic velocity occurring near the lumen centerline was extracted.

In cases of stenosis, the VFI framework occasionally produced unreliable flow velocity estimates due to complex, turbulent flow patterns and low signal-to-noise ratios in the post-stenotic region. Therefore, in all the stenosis cases considered in this study, the PINN model was adapted to rely solely on wall motion data. The model's ability to converge using wall-only data was previously verified in the *in silico* study under varying boundary conditions [39].

### Physics-Informed Neural Network (PINN) Model

#### Governing Equations

The PINN model employed in this study is based on the linearized one-dimensional equations governing pulse wave propagation in compliant, heterogeneous arterial segments. These equations, derived under the assumptions that the fluid velocity is much smaller than the pulse wave velocity and that wall inertial and viscous forces are negligible (valid for large vessels), are given by:

(1) $$k_{p}\frac{\partial P}{\partial t}+A\frac{\partial u}{\partial x}=0$$

(2) $$\frac{\partial u}{\partial t}+\frac{1}{\rho }\frac{\partial P}{\partial x}+K_{R}u=0$$

(3) $$A=A_{0}+k_{p}\left(P-P_{0}\right)$$

where P is the luminal blood pressure, u is the axial blood flow velocity, x is the spatial coordinate along the vessel, t is time, A is the lumen cross-sectional area, ρ is the blood density (assumed to be 1060 kg/m^3^), $K_{R}$ is the viscous resistance per unit length, and $k_{p}$ is the local arterial wall compliance. Equation (3) represents the Bramwell-Hill relation, linking changes in lumen area to changes in pressure via the compliance parameter. In this formulation, $A_{0}$ and $P_{0}$ are reference area and pressure values.

The compliance $k_{p}$ can also be expressed as:

(4) $$k_{p}=\frac{\Delta A}{\Delta P}$$

#### Neural Network Architecture

The PINN model was parameterized using a fully connected feedforward neural network with a multiscale Fourier feature mapping. This architecture is particularly suited for problems involving disparate spatiotemporal scales, such as pulse wave propagation, where temporal variations are much faster than spatial variations.

**Figure 1:**
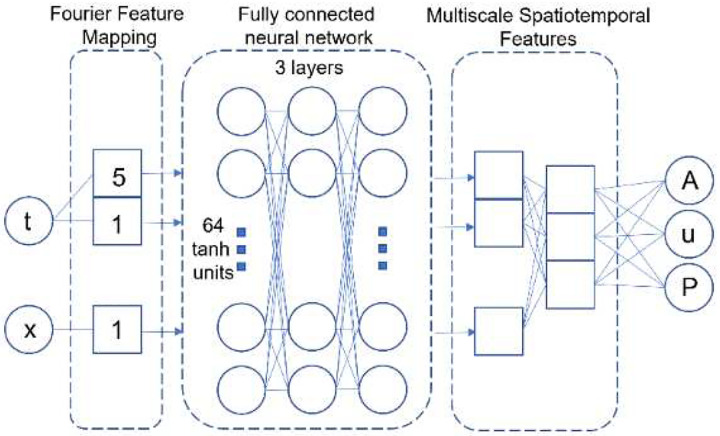
Schematic of the neural network architecture used in the proposed PINN model, consisting of an input layer for space–time coordinates, a Fourier feature mapping layer, three fully connected hidden layers with 64 neurons and tanh activation, a multiscale spatiotemporal feature layer, and an output layer predicting lumen area, flow velocity, and pressure.

The input layer of the network accepted two variables: the spatial coordinate x and the temporal coordinate t. These inputs were first transformed via a Fourier feature mapping to capture multiscale behavior. Specifically, Fourier basis functions with scales of were applied to the time dimension, while a scale of 1 was applied to the spatial dimension. The Fourier-mapped features were then passed through three hidden layers, each containing 64 neurons with hyperbolic tangent (tanh) activation functions. The output layer produced three quantities: the lumen cross-sectional area A(x,t), the axial flow velocity u(x,t), and the luminal pressure P(x,t). A schematic of the PINN architecture is provided in Figure 1.

To improve the numerical conditioning of the optimization problem and ensure that all terms in the loss function were of similar magnitude, the input and output variables were non-dimensionalized. The characteristic length scale was chosen as the square root of the mean cross-sectional area of the imaged arterial segment. The velocity was normalized by its peak value across the spatial and temporal domain. All inputs were further standardized to have zero mean and unit variance prior to training.

#### Loss Function

The neural network was trained by minimizing a composite loss function that combined two components: a data fidelity term and a physics residual term. The data fidelity term, $L_{\text{Data}}$, quantified the mismatch between the network's predictions and the measured wall motion and flow velocity data:

(5) $$L_{\text{Data}}=\frac{1}{N_{x}N_{t}}\sum_{i,j}{\left(\left[\overset{ˆ}{A}\left(x_{i},t_{j}\right),\overset{ˆ}{u}\left(x_{i},t_{j}\right)\right]-\left[A_{\text{meas}}\left(x_{i},t_{j}\right),u_{\text{meas}}\left(x_{i},t_{j}\right)\right]\right)}^{2}$$

where $N_{x}$ and $N_{t}$ are the number of spatial and temporal sampling points, respectively, and the hat notation denotes network predictions.

The physics residual term, $L_{\text{PDE}}$, enforced adherence to the governing equations (1) and (2):

(6) $$L_{\text{PDE}}=\frac{1}{N_{x}N_{t}}\sum_{i,j}\left[{\left(k_{p}\frac{\partial P}{\partial t}+A\frac{\partial u}{\partial x}\right)}^{2}+{\left(\frac{\partial u}{\partial t}+\frac{1}{\rho }\frac{\partial P}{\partial x}\right)}^{2}\right]$$

The partial derivatives in Equation (6) were computed using automated differentiation, a key feature of modern deep learning frameworks that enables efficient and exact gradient computation. The total loss function was then:

(7) $$L_{\text{total}}=w_{\text{Data}}L_{\text{Data}}+w_{\text{PDE}}L_{\text{PDE}}$$

where $w_{\text{Data}}=0.04$ and $w_{\text{PDE}}=100$ were weighting coefficients chosen to balance the two terms based on their typical magnitudes. These weights were held constant across all cases in this study, consistent with our prior *in silico* and phantom validation study [39].

#### Training Configuration and Model Outputs

The neural network was trained using the Adam optimizer, a gradient-based optimization algorithm well-suited for large-scale, noisy problems. The learning rate was set to 0.001, with a decay rate of 0.8 applied to facilitate convergence. Training was performed for a fixed number of 20,000 iterations, which was empirically determined to yield convergent solutions in the prior *in silico* and phantom study [39].

The training data consisted of the measured wall motion and flow velocity fields corresponding to one complete cardiac cycle was extracted from the multi-cycle acquisition, yielding approximately 2778 temporal samples (given the 3 kHz effective frame rate and a cardiac cycle duration of approximately 0.9 seconds). We considered the L7–4 ultrasound transducer in the data acquisition leading to a lateral field of view of approximately 40 mm. In the stenotic cases, since we need higher lateral resolution due to the localized variations of the geometry and compliance, we considered all the 128 elements of the L7–4 transducer resulted in a training data matrix of size 128 × 2778 for both wall motion and flow velocity. In the case of healthy subjects, because of the homogeneity of the geometry and material properties within the field of view, we downsampled the data by a factor of 8 to reduce the computational time while maintaining the accuracy.

All computations were performed on a standard desktop workstation equipped with an NVIDIA Quadro RTX 5000 GPU with 16 GB of RAM. Training times ranged from approximately 20 minutes for healthy subjects with down-sampled data to up to 1.5 hours for stenotic cases with high-fidelity data. Once the training was completed with fixed number of iterations of 30k, the converged outputs from the neural network are the spatiotemporal distributions of pressure P(x,t), velocity u(x,t), and lumen area A(x,t). The compliance field $k_{p}(x)$ is obtained after post-processing the converged outputs from the PINN model mentioned below. The compliance was parameterized as a spatially varying field with the same spatial resolution as the input data.

### Compliance and Derived Metrics

Once the PINN model converged, the optimized compliance field $k_{p}(x)$ was extracted by post-processing the changes of lumen area and pressure, $k_{p}=\Delta A/\Delta P$. This spatially resolved compliance map represents the inverse of arterial stiffness; lower compliance leads to higher arterial stiffness and vice versa.

In addition to compliance, the PINN model also provided spatiotemporal maps of luminal pressure P(x,t) and flow velocity u(x,t). The pressure waveform at the middle element (64^th^ out of the 128 elements) of the transducer (i.e., (x=18.9mm,t)) was extracted and compared qualitatively with physiological expectations of luminal blood pressure.

### Validation and Convergence Metrics

To assess the accuracy of the PINN model, the network's predictions of wall motion and flow velocity were compared with the measured data. The normalized difference was computed as:

(9) $$\text{Normalized difference}=\frac{\|\text{PINN output}-\text{Measured data}{\|}^{2}}{\|\text{Measured data}{\|}^{2}}$$

where ‖⋅‖ denotes the Euclidean (L2) norm. This metric quantified the goodness-of-fit for both the wall motion and flow velocity fields. Additionally, the convergence of the optimization was monitored by tracking the total loss, data loss, and PDE loss over the 20,000 training iterations. Convergence was deemed successful when the loss plateaued and the predicted wall motion and flow velocity closely matched the measured spatiotemporal data. While we considered fixed iteration of 20,000 in this study, an adaptive epochs number can be assigned depending on the rate at which the loss function is going down.

### Adaptations for *In Vivo* Human Data

This study is based on our prior work on *in silico* simulations and phantom experiments [39] to clinical data acquired in human subjects. Several key adaptations were necessary to accommodate the unique challenges of *in vivo* imaging:

1. Unknown Boundary Conditions: Unlike controlled phantom experiments, where inlet and outlet boundary conditions could be specified or measured, *in vivo* boundary conditions are unknown. The PINN approach circumvents this limitation by not requiring explicit boundary condition specification; the physics residual term in the loss function implicitly enforces consistency with the governing equations throughout the domain. The effect of the boundary conditions was studied extensively in our earlier studies where we varied the inlet and outlet boundary conditions in the *in silico* simulations [39].
2. Natural Cardiac Waveforms: The cardiac waveforms in human subjects are inherently more variable and complex than the programmable waveforms used in phantom studies. The PINN model was robust to this variability, as demonstrated by its ability to fit the data across multiple subjects with different heart rates and waveform morphologies.
3. Surrounding Tissue: *In vivo* imaging occurs in the presence of surrounding soft tissue, which can introduce acoustic aberration and attenuation. Our prior phantom study [39] validated the model's robustness to background tissue by incorporating ultrasound scatterers (graphite) in the surrounding gelatin, confirming minimal impact on compliance estimation.
4. Patient-Specific Anatomy and Pathology: Each subject exhibited unique anatomical features and, in the case of stenotic patients, varying degrees and morphologies of anatomical variation. The spatially resolved compliance estimation afforded by the PINN model enabled localization of stiffness changes corresponding to visible anatomical changes on the B-mode images.
5. Unreliable VFI in Stenotic Cases: In regions of stenosis, the VFI framework occasionally produced unreliable flow velocity estimates due to turbulent flow and low signal-to-noise ratios. The PINN model was designed to handle missing or poor-quality flow data by down-weighting or excluding the VFI data from the loss function in affected regions. In the stenosis cases considered in this study, the model relied primarily on wall motion data, leveraging the robustness to incomplete data previously demonstrated in the *in silico* study [39].
6. Computational Feasibility: Despite the increased complexity of clinical data, the computational time remained clinically feasible, ranging from 20 minutes (for down sampled data in the cases of healthy subjects) to 1.5 hours (for data from all the elements of the transducer in the cases of stenotic subjects) per case on standard desktop machine with GPU (Quadro RTX 5000 with 16 GB memory) hardware. Future optimization of the model and hardware could further reduce this time toward real-time or near-real-time applications.

## Results

### Healthy Subject Case

**Figure 2:**
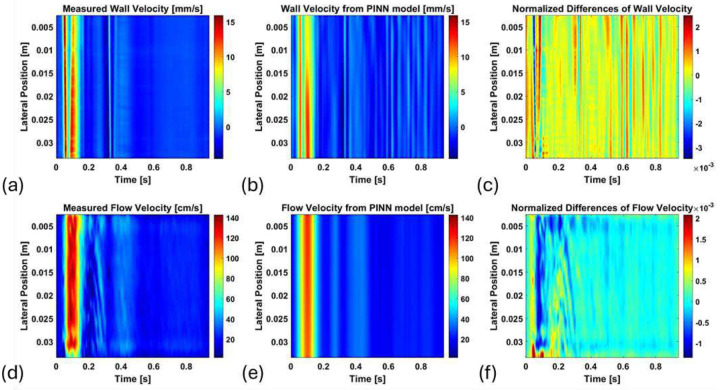
Comparison of the measured data from the PWI and VFI framework with the converged solution from the PINN framework corresponding to the healthy individual (case #1 in Table 1). (a)–(c) Measured arterial wall motion, PINN-predicted wall motion, and their difference respectively. (d)–(f) Measured fluid velocity, PINN-predicted velocity, and their difference respectively.

Results in the healthy individual (Subject #1, in Table 1) are presented first, followed by analyses of the carotid stenosis cases. Figures 2 (a) and (d) display the measured wall velocity and flow velocity results acquired via the combined PWI and VFI framework. The corresponding PINN model outputs are shown in Figure 2 (b) and (e), with normalized differences computed using Equation 9 presented in Figure 2 (c) and (f). The maximum % difference between measured and PINN-predicted values was under 0.2% for both wall velocity and flow velocity, indicating agreement between the model predictions and measured data.

**Figure 3:**
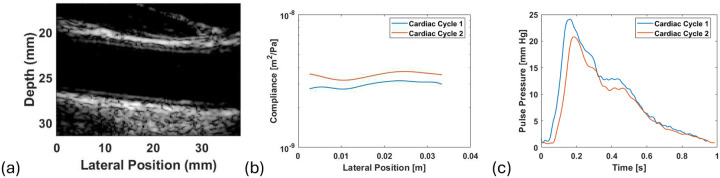
(a) B-mode ultrasound image of the common carotid artery (CCA) corresponding to the healthy individual (case #1 in Table 1). (b) Estimated spatially-resolved arterial compliance from the proposed model. (c) PINN output showing pulse pressure. Blue and red lines in (b) and (c) represent the outputs corresponding to the two successive cardiac cycles to show repeatability.

The B-mode ultrasound image of the common carotid artery (Figure 3 (a)) demonstrates uniform arterial anatomy without any visible atherosclerotic accumulation. The spatially resolved compliance field estimated by the PINN model is presented in Figure 3 (b), showing homogeneous compliance distribution along the imaged arterial segment. The luminal pressure waveform extracted at the middle element (64th of 128 elements of the considered L7–4 ultrasound transducer; spatial position x = 18.9 mm) is shown in Figure 3 (c). Both the compliance and pressure waveforms exhibited repeatability across two successive cardiac cycles (blue and red curves), demonstrating the robustness and consistency of the PINN framework under physiologic conditions.

For comparison, the Moens-Korteweg equation [31] was applied to estimate global compliance and pulse wave velocity, yielding values of 1.3 × 10^−9^ m^2^/Pa and 4.18 m/s, respectively. The corresponding PINN-derived values, computed as spatial averages across the arterial segment, were 2.98 × 10^−9^ m^2^/Pa and 3.68 m/s. Here it should be noted that, the Moens Korteweg derived values do not correct compliance (or pulse wave velocity) estimates for both anatomical and flow effects. In contrast, the PINN model derived quantities are estimated by considering both anatomical and flow effects through its integration of governing hemodynamic equations with measured spatiotemporal data.

### Carotid Stenosis Cases

#### Subject #2 (Proximal Common Carotid Artery)

**Figure 4:**
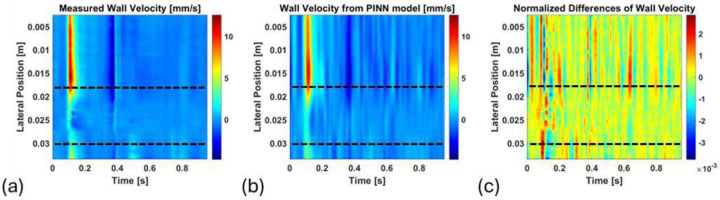
Comparison of the measured data from the PWI framework with the converged solution from the PINN framework corresponding to the carotid stenosis patient (case #2 in Table 1). (a)–(c) Measured arterial wall motion, PINN-predicted wall motion, and their difference respectively. The region between the black dashed lines indicates the morphological changes on the corresponding B-mode image.

Analysis of the first stenosis case (Subject #2, in Table 1) revealed measured wall velocity, PINN-predicted wall velocity, and their normalized differences in Figures 4 (a) to (c) respectively. The maximum normalized percentage difference in wall velocity between the measured and PINN-predicted wall velocity was 0.35%, confirming model convergence. In sharp contrast to the healthy case mentioned in the above subsection, blood flow velocity data were excluded from the PINN framework for all stenosis cases due to signal degradation in stenotic regions. The measured flow velocity data for Subjects #2 and #3 are presented in Figure 6, showing substantial noise and missing data consistent with complex (turbulent) flow patterns distal to the stenosis.

**Figure 5:**
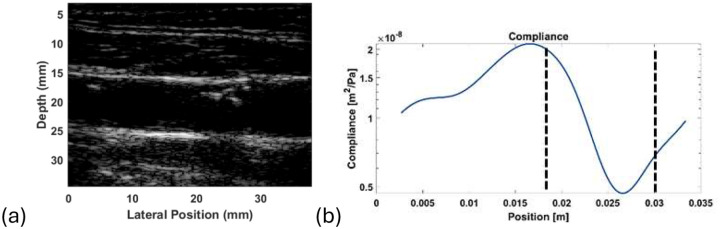
(a) B-mode ultrasound image of the common carotid artery (CCA) corresponding to the carotid stenosis patient (case #2 in Table 1). (b) Estimated spatially-resolved arterial compliance from the proposed model. The region between the black dashed lines indicates the morphological changes on the corresponding B-mode image.

The B-mode image (Figure 5 (a)) depicts changes in arterial geometry and/or echogenicity beginning approximately at 15 mm from the lateral edge of the field of view. The estimated compliance map (Figure 5 (b)) reflects this anatomical variation, consistent with the B-mode image. This spatial correspondence between ultrasound morphology and PINN-estimated mechanical properties validates the model's ability to identify the stiffness changes.

#### Subject #3 ((Distal Common Carotid Artery)

The second stenosis patient (Subject #3, in Table 1) was analyzed similarly. Measured, PINN-predicted wall motion and the corresponding normalized differences are presented in Figure 7 (a) to (c), with a maximum percentage difference of 0.3% in wall velocity. The B-mode image (Figure 8 (a)) reveals material accumulation toward the lateral direction of the distal common carotid artery. The corresponding compliance map (Figure 8 (b)) exhibits localized variations consistent with the observed plaque morphology, further demonstrating the model's capacity to map spatially heterogeneous arterial stiffness in stenotic vessels.

#### Subject #4 (Distal Common Carotid Artery and Carotid Bifurcation)

This subject (Subject #4, in Table 1) presented imaging that captured the transition region between the distal CCA and the carotid bifurcation. Figure 9 (a)–(c) show the measured, PINN-predicted wall velocities, and the corresponding normalized differences respectively, with a maximum normalized percentage difference of 0.48%, the largest deviation observed in this study. The B-mode image (Figure 10 (a)) reveals the location of the bifurcation on the distal side of the field of view, and the corresponding compliance map (Figure 10 (b)) shows spatially varying compliance changes correlating with the anatomical transition.

#### Subject #5 (Distal Common Carotid Artery and Carotid Bifurcation)

Figure 11 (a) to (c) present the measured, PINN-derived wall velocity and the corresponding normalized differences respectively. Here, the maximum % difference is 0.35%. The B-mode image (Figure 12 (a)) shows the beginning of the bulb region on the lateral side of the field of view. The estimated compliance map (Figure 12 (b)) shows a corresponding drop in compliance (higher stiffness) at this location, confirming the spatial correlation between estimated mechanical properties and anatomical transition.

Channel data was acquired from an additional imaging site in the same subject mentioned in the above paragraph, positioned further distally and capturing the bulb region in the middle of the field of view. Figure 13 (a) to (c) present the measured, PINN-predicted wall velocity, and the corresponding normalized differences respectively, with a maximum percentage difference of 0.42%. The B-mode image (Figure 14 (a)) shows the anatomical transition corresponding to the bifurcation region, which is also reflected in the estimated compliance map (Figure 14 (b)). The estimated compliance varies spatially in accordance with the anatomical feature, demonstrating the model's ability to detect localized stiffness variations in different arterial segments of the same subject.

## Discussion

This study presents a novel approach to characterizing arterial mechanical properties through spatially-resolved compliance estimation, with the integration of physics-informed neural networks (PINNs) that synergistically combine both physics-driven and data-driven methodologies. This hybrid framework represents an advancement over traditional approaches that rely on either purely physics-based models or purely data-driven techniques, enabling more robust and adaptable analysis of complex arterial geometry and hemodynamics.

**Figure 6:**
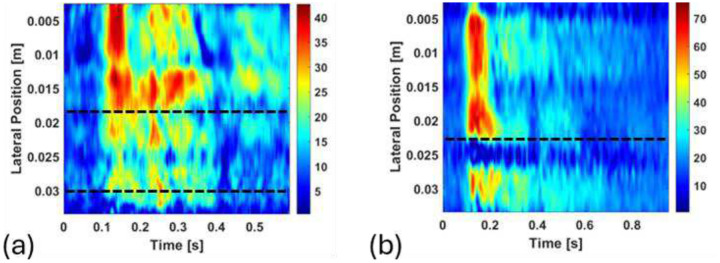
Measured blood flow velocity data from the VFI framework corresponding to the carotid stenosis patients #2 in (a) and #3 in (b). The details of these two patients are mentioned in Table 1. The region between the black dashed lines indicates the morphological changes on the corresponding B-mode image.

The reconstruction accuracy of the proposed PINN model demonstrates promising performance, with expected limitations in stenotic cases attributable to inherent model constraints and assumptions. Specifically, the flow velocity data from the vector flow imaging (VFI) framework exhibits missing and noisy components, as illustrated in Figure 6, resulting from the turbulent post-stenotic flow patterns inherent to diseased vessels. Current efforts are focused on refining the VFI framework through parameter optimization and exploration of alternative methodologies. In the present iteration of the PINN model applied to stenotic arteries, we hence employed only wall motion data and restricted the compliance estimation process to this single input modality (wall motion data). Prior phantom validation studies [39] demonstrated a 13.84% improvement in compliance accuracy when integrating both wall motion and flow velocity data compared to wall-only approaches. Notably, the current study revealed that the model maintained clinically acceptable performance using wall motion data alone, despite significant anatomical complexity. This adaptive degradation capability is particularly valuable for clinical translation, as image quality is frequently compromised in stenotic, heavily calcified, or tortuous arterial segments. Rather than producing unreliable estimates due to data scarcity, the PINN model maintains utility in challenging clinical scenarios.

The estimated compliance correlated with anatomical variations observed in healthy individuals (Figure 2 and Figure 3), providing additional evidence for model validity. The PINN framework simultaneously estimated luminal blood pressure (Figure 3 (c)) throughout the cardiac cycle without requiring invasive pressure measurements or explicit boundary condition specification. The reconstructed pressure waveforms exhibited physiologically realistic characteristics, including appropriate systolic and diastolic phases, pulse pressure magnitudes, and temporal dynamics consistent with expected carotid artery hemodynamics. Although direct validation against invasive or calibrated non-invasive pressure measurements was unavailable in this preliminary feasibility study, the qualitative agreement with established physiologic principles provides encouraging preliminary evidence for the model's applicability to pressure estimation. Future investigations incorporating calibrated applanation tonometry or other established non-invasive pressure reference techniques would substantially enhance confidence in the estimated pressure fields. Additionally, excellent repeatability was observed between successive cardiac cycles for both compliance (Figure 3 (b)) and luminal pressure (Figure 3 (c)) estimates, indicating temporal consistency and model stability.

Examination of the carotid stenosis subject (case #2 in Table 1) revealed that estimated compliance similarly follows the anatomical variation captured in corresponding B-mode ultrasound images, as detailed in Figure 5. Although histological characterization of plaque composition was unavailable for this case, acoustic shadowing evident in the B-mode image beneath the plaque region (Figure 5 (a)) indicates a stiffer tissue composition. Correspondingly, the spatially-resolved compliance map (Figure 5 (b)) demonstrates reduced compliance at the lateral location exhibiting acoustic shadowing, validating the model's sensitivity to mechanical property variations.

**Figure 7:**
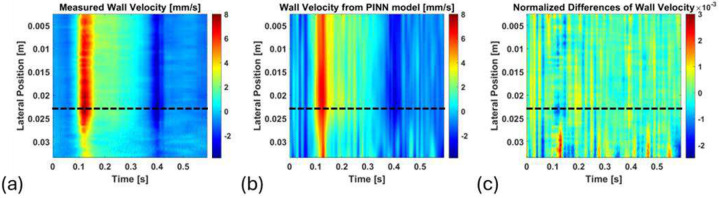
Comparison of the measured data from the PWI framework with the converged solution from the PINN framework corresponding to the carotid stenosis patient (case #3 in Table 1). (a)–(c) Measured arterial wall motion, PINN-predicted wall motion, and their difference respectively. The black dashed line demarcates the region of the morphological changes on the corresponding B-mode image.

**Figure 8:**
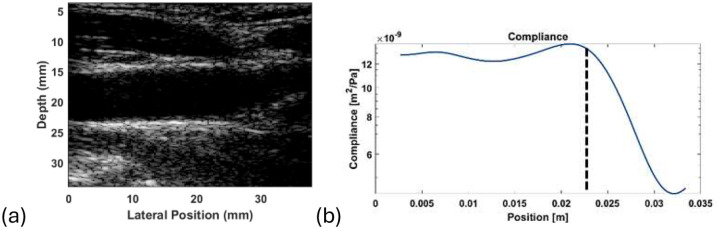
(a) B-mode ultrasound image of the common carotid artery (CCA) corresponding to the carotid stenosis patient (case #3 in Table 1). (b) Estimated spatially-resolved arterial compliance from the proposed model. The black dashed line demarcates the region of the morphological changes on the corresponding B-mode image.

To establish the effectiveness of the proposed model across multiple stenotic cases, we examined an additional patient (Figure 7 and Figure 8), with observations consistent with the previously described case, further supporting the generalizability of the approach.

**Figure 9:**
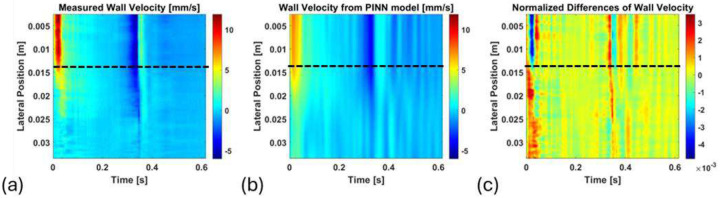
Comparison of the measured data from the PWI framework with the converged solution from the PINN framework corresponding to the carotid stenosis patient (case #4 in Table 1). (a)–(c) Measured arterial wall motion, PINN-predicted wall motion, and their difference respectively. The black dashed line demarcates the region of the morphological changes on the corresponding B-mode image.

**Figure 10:**
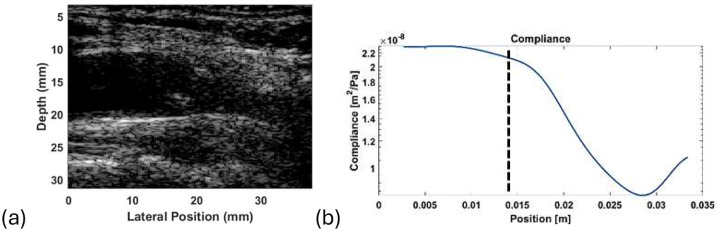
(a) B-mode ultrasound image of the common carotid artery (CCA) corresponding to the carotid stenosis patient (case #4 in Table 1). (b) Estimated spatially-resolved arterial compliance from the proposed model. The black dashed line demarcates the region of the morphological changes on the corresponding B-mode image.

In a subsequent case presented in Figure 9 and Figure 10, analysis focused on the very distal common carotid artery where the carotid bifurcation region begins. The PINN model reconstructed wall motion with acceptable accuracy, although the percentage difference (0.48%) was elevated relative to other cases in the study population. This elevated error is expected, as the bifurcation geometry and complex flow patterns violate several key assumptions embedded in the underlying partial differential equations governing arterial mechanics. Nevertheless, the estimated compliance map (Figure 10 (b)) follows the same spatial patterns observed in previous cases, demonstrating anatomical fidelity with respect to the B-mode ultrasound image (Figure 10 (a)). Notably, the bifurcation region exhibited increased stiffness (reduced compliance), a finding consistent with observations from prior investigations in the literature [40].

**Figure 11:**
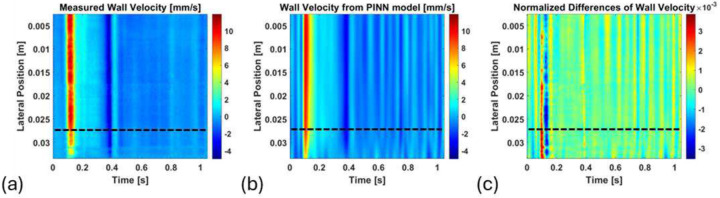
Comparison of the measured data from the PWI framework with the converged solution from the PINN framework corresponding to the carotid stenosis patient (case #5 in Table 1). (a)–(c) Measured arterial wall motion, PINN-predicted wall motion, and their difference respectively. The black dashed line demarcates the region of the morphological changes on the corresponding B-mode image.

**Figure 12:**
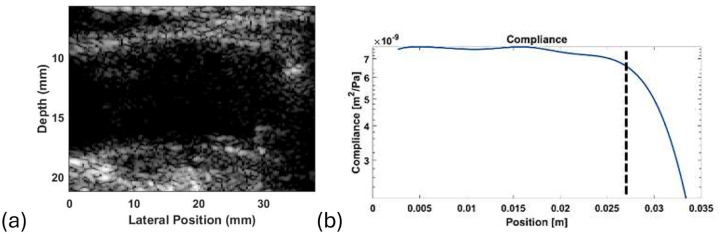
(a) B-mode ultrasound image of the common carotid artery (CCA) corresponding to the carotid stenosis patient (case #5 in Table 1). (b) Estimated spatially-resolved arterial compliance from the proposed model. The black dashed line demarcates the region of the morphological changes on the corresponding B-mode image.

**Figure 13:**
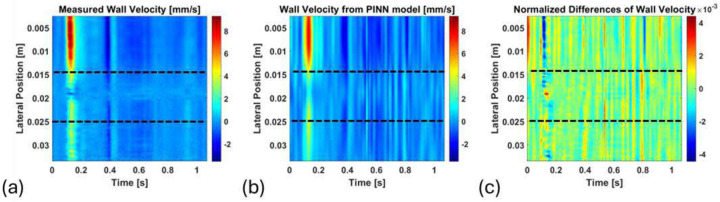
Comparison of the measured data from the PWI framework with the converged solution from the PINN framework corresponding to the carotid stenosis patient (case #5 in Table 1). This is the same subject case mentioned above in Figure 11 and Figure 12, but the imaging location is further distally and capturing the bulb region in the middle of the field of view. (a)–(c) Measured arterial wall motion, PINN-predicted wall motion, and their difference respectively. The region between the black dashed lines indicates the morphological changes on the corresponding B-mode image.

**Figure 14:**
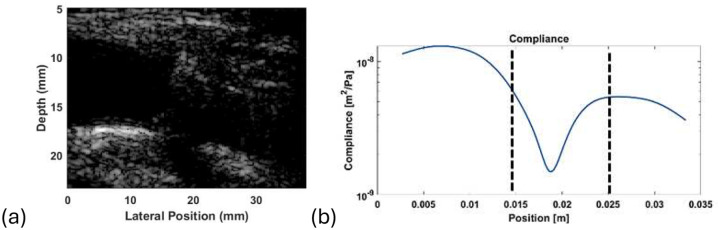
(a) B-mode ultrasound image of the common carotid artery (CCA) corresponding to the carotid stenosis patient (case #5 in Table 1). This is the same subject case mentioned above in Figure 11 and Figure 12, but the imaging location is further distally and capturing the bulb region in the middle of the field of view. (b) Estimated spatially-resolved arterial compliance from the proposed model. The region between the black dashed lines indicates the morphological changes on the corresponding B-mode image.

Analysis of an additional patient (patient #5 in Table 1) included examination of both the bifurcation transition region (Figure 11 and Figure 12) and imaging configurations with the bulb positioned centrally within the field of view (Figure 13 and Figure 14). In both scenarios, the estimated compliance spatial variation remained consistent with the morphology on the B-mode ultrasound. Furthermore, both configurations demonstrated reduced compliance (increased stiffness) in the bulb region, confirming the pattern observed in previous case and consistent with published observations [40]. Similar to the preceding bulb case, percentage differences between measured and PINN-converged wall velocity (0.35% and 0.42% for the two imaging orientations) were elevated compared to healthy cases and stenotic pre-plaque regions, reflecting violations of the underlying physics model assumptions inherent to the PINN framework. Here note that we kept the number of epochs same across all the cases considered in this study. We observed similar discrepancy in the post-stenotic region in our prior phantom studies [39], providing additional confirmation. However, despite imperfections in the physics-based component of the model, the data-driven segment of the PINN architecture, particularly the neural network structure, compensates for deficiencies in the physics representation. This complementary relationship between physics-driven and data-driven approaches constitutes the fundamental advantage of the proposed framework.

Model optimization and refinement efforts should address several technical considerations. The current PINN implementation employs manually-adjusted weight coefficients for individual loss function components (100 for physics-derived loss and 0.04 for data-driven loss), selected based on numerical magnitude of respective terms. These weighting parameters are critical for balancing physics and data-driven contributions across varying case complexities. Future work should explore systematic optimization or adaptive tuning of these weights, treating them as unknown parameters within the inverse problem framework. Additionally, early stopping criteria could be implemented by monitoring the convergence rate of the total loss function.

Beyond PINN-specific refinements, the underlying physics model can be substantially advanced through dimensional expansion from one-dimensional to higher-dimensional formulations (2D/3D) and incorporation of higher-order physical phenomena. Specifically, incorporating nonlinear constitutive models to characterize hyperelastic arterial behavior and viscoelastic formulations to simulate both elastic and viscous properties of arterial wall tissues would enhance biophysical fidelity. Furthermore, integration of high-fidelity hemodynamic models within the physics-driven PINN component could better simulate turbulent flow patterns in post-stenotic regions.

Assessment of absolute accuracy presents inherent constraints in this *in vivo* investigation. While the PINN framework successfully produced anatomically consistent compliance maps and physiologically plausible pressure waveforms, rigorous validation of absolute accuracy would require either invasive pressure measurements or *ex vivo* mechanical testing of arterial specimens, procedures neither feasible nor ethically justified in a clinical feasibility study. Our previous phantom investigations [39] provided partial validation against known material properties, yet inherent differences between polyvinyl alcohol (PVA) phantom materials and native arterial tissue, including nonlinearity, viscoelasticity, and three-dimensional architectural complexity, limit direct extrapolation of phantom accuracy to *in vivo* performance.

The PINN-based ultrasound approach occupies a complementary position within the broader landscape of established plaque characterization methodologies. Magnetic resonance imaging (MRI) excels at detecting intraplaque hemorrhage and lipid content but is characterized by substantial time and cost requirements. Intravascular ultrasound (IVUS) elastography delivers exceptional spatial resolution but requires vascular access through invasive procedure. The non-invasive, relatively expeditious ultrasound-based PINN approach could serve as a pre-screening instrument, identifying high-risk patients warranting further investigation via MRI or IVUS, or as a longitudinal monitoring tool tracking mechanical property evolution in patients with established plaques. Computational efficiency is clinically relevant, with processing times ranging from 20 minutes to 1.5 hours per subject using standard GPU hardware, rendering the approach feasible for offline analysis concurrent with clinical ultrasound examinations, though not yet suitable for real-time on-probe implementation. With continued hardware acceleration and algorithmic refinement, the methodology could reasonably transition toward rapid or interactive clinical workflows, wherein compliance and pressure mapping could be generated within minutes of data acquisition.

## Conclusions

This study demonstrates the clinical feasibility of a physics-informed neural network (PINN) framework for noninvasive, spatially resolved estimation of arterial compliance and luminal pressure from high-frame-rate ultrasound data in *in vivo* human subjects. The PINN approach overcame limitations of missing or unreliable flow data, without requiring boundary condition specification. Spatial compliance (inverse of stiffness) variations corresponded with atherosclerotic plaques and/or anatomical transitions identified by the ultrasound B-mode images, highlighting its utility for localized vascular disease assessment. Computational times were clinically practical, supporting potential integration into diagnostic workflows. While validation in larger and more diverse patient cohorts is needed, these results provide a promising new avenue toward personalized vascular mechanics characterization and improved risk stratification of atherosclerotic plaques. Future work would focus on expanding model dimensionality, integrating nonlinear tissue properties, and conducting prospective outcome studies for broader clinical adoption.

## Figures and Tables

**Table 1:** Considered study cohort comprising of one healthy subject and four carotid stenosis patients

Subject No.	Sex	Age	Notes
**1**	Male	25	Healthy case
**2**	Male	60	Stenosis level: <50% for both left and right CCA
**3**	Female	78	Stenosis level: <50% for both left and right CCA
**4**	Female	74	Stenosis level: >50% left CCA, and <50% for right CCA
**5**	Male	81	Stenosis level: <50% left CCA, and 50–69% right CCA

## Data Availability

The data supporting the findings of this study will be made available upon request. Please contact the corresponding author for access to the datasets used in this research.
